# Sustained neutrophil infiltration and bacterial grain morphology underlie chronic mycetoma pathology in a murine model

**DOI:** 10.22038/ijbms.2025.87349.18875

**Published:** 2025

**Authors:** Maria L. Ruiz-de la Cruz, Anna V. Vazquez -Marmolejo, Manuel G. Mejia-Torres, aria de los Angeles Castro-Corona, Mario C. Salinas-Carmona

**Affiliations:** Department of Immunology, School of Medicine and Dr. Jose Eleuterio Gonzalez University Hospital, Universidad Autonoma de Nuevo León, Monterrey, Mexico

**Keywords:** Animal disease models, Granuloma, Host-pathogen interactions, Immune dysregulation, Neutrophil infiltration, Nocardia infections

## Abstract

**Objective(s):**

This study aimed to characterize the progression of chronic mycetoma caused by *Nocardia brasiliensis* in a BALB/c murine model, focusing on the interplay between host cellular immune responses, bacterial burden, and histopathological evolution.

**Materials and Methods:**

BALB/c mice were inoculated with *N. brasiliensis* in the left hind footpad to establish the mycetoma model. The mice were divided into four experimental groups: 0, 70, 100, and 365 days post-infection (dpi). Lesion volume was assessed throughout the course of infection. At the defined time points, bacterial load (serial dilution method), percentages of immune cell populations (flow cytometry), serum cytokines (interleukins IL-6, IL-10, and IL-12p70, monocyte chemoattractant protein-1 (MCP-1), interferon-gamma (IFN-γ), and tumor necrosis factor (TNF)) via cytometric bead array (CBA), as well as histopathology and bacterial grain morphology (H&E staining), were evaluated.

**Results:**

Chronic mycetoma progression revealed stable bacterial burden and lesion volume stabilization after 70 dpi through 365 dpi. Systemic expansion of CD4+ T cells in the spleen and sustained neutrophil dominance (>90% infiltration) characterized chronic lesions. Progressive tissue necrosis and panniculitis, undetectable by external lesion size, emerged histologically. Serum IL-6 levels surged during chronicity, suggesting a Th17 polarization, contrasting with declining MCP-1. Bacterial grains transitioned from club-shaped to circular over time, suggesting structural grain remodeling.

**Conclusion:**

In chronic experimental mycetoma, the cell response is mainly characterized by neutrophil infiltration, an altered CD4+ T cell response, and dysregulated cytokine production. The shape of bacterial grains continues to change, and the bacterial load remains constant.

## Introduction

Mycetoma is a chronic granulomatous infectious disease that affects the skin and subcutaneous tissues. Clinically, it is identified by three main signs: the development of tumorous swellings, the formation of draining sinus tracts, and the secretion of infectious grains that harbor the causative pathogen (1).

This chronic infection is listed as a neglected tropical disease by the World Health Organization. It is endemic in Africa, Asia, and Latin America regions, particularly affecting rural populations, leading to disability, social stigma, and economic hardship (2). Despite ongoing efforts to engage affected populations and effectively manage this condition, the delayed clinical recognition, often occurring decades after the initial signs of infection, leads to the progression of lesions into chronic stages (3), which often presents as recalcitrance to treatment and irreversible tissue damage (4). This underscores the need for a deeper understanding of the long-term host immune system and pathogen dynamics associated with mycetoma progression.

Research has shown that innate immune cells are unable to clear infections from virulent strains of *Nocardia spp.* on their own. This limitation stems from the bacteria’s effective resistance to cells’ mechanisms, including polymorphonuclear cell (PMN) responses, such as catalase and superoxide dismutase activity, which prevent the oxidative burst, as well as non-oxidative mechanisms, including neutrophils’ primary granules (5). Additionally, the cord factor or trehalose-6,6’-dimycolate inhibits phagosome-lysosome calcium-dependent membrane fusion in macrophages (6, 7), while *Nocardia* membrane lipids trigger a transient granulomatous reaction (8). Monocytes pre-stimulated with recombinant type 1 T helper (Th1) cytokines, TNF-α, and IFN-γ also prove ineffective at controlling *Nocardial* growth (9). This ineffectiveness may be associated with the heightened synthesis of lysosomal hydrolases, such as acid phosphatase, which bacteria can utilize as a carbon source (10). Furthermore, studies in adaptive immune effector cells confirm that T cells play a crucial role in managing infections caused by this bacterium. Unlike B cells, which are dispensable in bacterial containment, *Nocardia*-primed T cells exhibit the ability to directly kill *Nocardia spp.,* in contrast to unstimulated lymphocytes (11, 12). 

Cytokines serve as pivotal orchestrators of both innate and adaptive immune responses, critically shaping granulomatous inflammation and the host’s ability to control intracellular pathogens. In mycetoma, IL-6 and TNF-α drive neutrophilic recruitment and granuloma integrity (13, 14), mechanisms essential for containing *Nocardia* yet paradoxically contributing to tissue damage (15). IFN-γ and IL-12p70, central to Th1 polarization, enhance macrophage antimicrobial pathways often subverted in mycetoma (16, 17). Conversely, IL-10 promotes an immunosuppressive milieu that favors bacterial persistence, while declining MCP-1 (CCL_2_) reflects impaired monocyte recruitment—a hallmark of failed resolution in chronic infection (18–20). Together, these mediators may have a dual role in immunity to mycetoma, balancing containment and collateral damage. Murine models are fundamental in investigating mycetoma as the lesions progress in a manner that mimics human disease, thereby providing essential insights into cellular immune responses (21). However, existing studies predominantly focus on early infection stages, as the 90-day post-infection (dpi) stage is the longest studied, leaving the rest of the chronic phase uncharacterized (22, 23). This gap is critical, as the unique host-pathogen interactions remain unmodeled in preclinical studies.

Thus, the cellular immune response, both locally and systemically during the chronic phase of mycetoma induced by *Nocardia brasiliensis* in BALB/c mice, was examined, with observations extending up to one year post-infection. It is proposed that chronic mycetoma represents a pathological “steady state,” in which apparent clinical stability masks the underlying immunological and microbiological adaptations. 

## Materials and Methods

### Animals and ethical statement

Twenty-four female BALB/c mice, ten- to fourteen-week-old, weighing 20±2 g, were utilized in the present study. These animals were originally sourced from a colony generously provided by Carl Hansen (Small Animal Section, Veterinary Resources Branch, National Institutes of Health, Bethesda, MD, USA) and were maintained and processed under Biosecurity level 2 conditions, ensuring regulated temperature of 19-21°C, twelve-hour light/dark cycles, and controlled room humidity 60% ± 5, under specific pathogen-free conditions. Mice had ad libitum access to sterile water and Formulab Diet 5008 rodent chow (LabDiet, USA). Before inclusion in the study, mice underwent routine health monitoring conducted by veterinary personnel. Animal care and infection protocols were formulated following Mexican regulations (NOM-062-ZOO-1999) and international standards for animal welfare, including the National Institutes of Health Guide for the Care and Use of Laboratory Animals. The protocol received approval from the Institutional Animal Care and Use Committee (CICUAL) under registration number IN22-00004. 

### Experimental model of mycetoma caused by N. brasiliensis

Experimental actinomycetoma was induced by inoculating the footpads of mice using the previously reported protocol (24). Briefly, a suspension containing 40 mg (wet weight) of *N. brasiliensis* ATCC HUJEG-1 700358, obtained from a six-day culture in brain-heart infusion broth, was injected into the left hind footpad in a volume of 50 µl of sterile saline solution. Mice were euthanized at designated time points, and tissues were collected for analysis at 70, 100, and 365 days post-infection; a group of healthy controls, free from infection (designated as 0 dpi), was included in the study, having a total of n=6 per group of study. Animals were assessed for clinical signs of inflammation and lesion volume using a digital caliper, employing the ellipsoid formula (4/3π abc) to quantify the results in cubic centimeters, which serves as a measure of the lesion volume; for calculating the percentage in volume change in the baseline volume was established at0 dpi. Additionally, changes in mice weight were recorded in grams using a digital scale.

### Blood, spleen, lymph node, and infected tissue sampling

Mice were anesthetized with an intraperitoneal injection of 200 μl of a ketamine (Anesket, PISA Agropecuaria, Mexico) and xylazine (Procin, PISA Agropecuaria, Mexico) mixture, comprising 2 mg and 0.4 mg of ketamine and xylazine, respectively. Following anesthesia, euthanasia was performed via cervical dislocation. A cardiac puncture was performed to collect approximately 1 ml of blood. The spleen, ipsilateral popliteal lymph node, and the entire mycetoma, excised by making an incision at the junction between the tibia and the femur, were subsequently harvested for further experimental procedures.

### Tissue cell suspension

Spleen and popliteal lymph node were surgically removed, weighted on a digital scale, and placed in complete RPMI medium (cRPMI; RPMI 1640 supplemented with 2 mM L-glutamine, 1 mM sodium pyruvate, 10 mM HEPES, 1% nonessential amino acids, 100U of penicillin/ml, 100 µg of streptomycin/ml, and 10% fetal bovine serum; SLM-240, Sigma-Aldrich, USA). Tissue was gently disrupted mechanically using toothed forceps and gently pushed through a 70 µm cell strainer (Falcon, Corning, USA). Red blood cells were lysed with red blood cell lysing solution 1x (NH_4_Cl 8.02 g, NaHCO_3_ 0.84 g, EDTA disodium 0.37g, Millipore water quantity sufficient to (qs) 100 ml, for 10X solution) in agitation for ten minutes, washed and resuspended in cRPMI. The total cell numbers per tissue were determined manually by using a hemocytometer. 

The mycetoma tissue was mechanically disrupted as described above and therefore incubated with 5 ml cRPMI containing a mix of collagenase and hyaluronidase 1X (182.6 mg collagenase (C9891-16; Sigma-Aldrich, USA), 62.4 mg hyaluronidase (H3506-16; Sigma-Aldrich, USA) and qs 50 ml MEM, HBS medium (11575032; Gibco, USA ) for a 10x solution), for 30 min at 37 °C for enzymatic digestion. Washed with cRPMI, strained, lysed, resuspended, and counted as described above.

### Assessment of bacterial load of N. brasiliensis

Bacterial loads in the footpads of mice were quantified by performing serial dilutions of the cell suspension, which were plated in triplicate on brain heart infusion agar (241830; BD Difco, USA). After a one-week incubation period at 37 °C, *N. brasiliensis* colonies were counted, allowing for the calculation of colony-forming units (CFU) per gram of tissue.

### Fluorescent labeling of cells and gating strategy for flow cytometry

Cell suspensions derived from the three tissues, each containing 10^6^ cells/ml from individual mice, were incubated with monoclonal antibodies for flow cytometry, sourced from eBioscience (USA). These cells were stained for cell surface markers for lymphocytic population identification by recognizing CD3 (APC-Cy7 clone 17A2), CD4 (Pacific Blue clone RM4-5), and CD8a (V500 clone 53-6.7, and for myeloid cell populations on a separate tube were used surface markers for CD45 (BV421 clone I3/2.3), Ly6G (APC-Cy7 clone 1A8), and F4/80 (AF488 clone T45-2342) were used and incubated for 30 min at 4 °C in the dark, washed and resuspended in 500 µl of cRPMI. A total of 100,000 events per sample were acquired utilizing an LSR Fortessa cytometer, and the gating strategy was manually generated using fluorescence minus-one controls for identification of leucocyte populations with the FACSDiva software version 8.0 (BD Biosciences, USA). 

Lymphocytes were gated using a singlet selection in a forward scatter height (FSC-H) versus forward scatter area (FSC-A) gate, followed by using FSC-A versus side scatter area (SSC-A) gate. CD3+ (T cells) were identified within the mononuclear cell population, and the percentages of CD4+ helper T cells and CD8+ cytotoxic T cells were calculated relative to the total CD3+ cells. For myeloid analysis, CD45+ leukocytes were gated from singlets and then analyzed by using FSC-A vs SSC-A. Neutrophils were identified as Ly6G+ cells, while macrophages were defined as F4/80+ cells. Percentages were expressed relative to the total CD45+ leukocytes. Fluorescence minus-one controls were used to define populations positive to the mentioned markers.

### Multiplex serum cytokines analysis

The assay was conducted using serum from blood obtained via cardiac puncture at specified time intervals. The methodology outlined in the BD Cytometric Bead Array (CBA) Mouse Inflammation Kit (BD 552364; BD Biosciences, USA) was strictly adhered to. Cytokine quantification was performed using the BD LSR Fortessa cytometer, with FACSDiva software version 8.0 (BD Biosciences, USA) employed for analysis. Data analysis was executed using CBA Analysis Software (BD Biosciences) to determine the concentration of each cytokine, interleukins IL-6, IL-10, and IL-12p70, C-C motif chemokine ligand 2 (CCL2), also known as MCP-1, IFN-γ, TNF, in picograms per milliliter (pg/ml), referencing a standard curve derived from commercially available standards spanning concentrations from 20 to 5,000 pg/ml.

### Histopathology of infected tissue

A tissue biopsy from the infected footpad, with minimum dimensions of 0.5 cm x 0.5 cm, was dehydrated and embedded in paraffin following standard procedures. Tissue blocks were sectioned into 4 µm slices and mounted on slides for hematoxylin and eosin staining (H&E). Two board-certified pathologists, blinded to the experimental groups, evaluated the lesions in eight fields and assigned a semi-quantitative scoring system based on the following criteria: absence of the feature (none), scant presence (+), moderate presence (++), and abundant presence (+++). The features evaluated comprehended cell infiltration, including PMN cells, lymphocytes, plasma cells, and foamy cells; tissue lesions such as edema, vascular congestion, necrosis, panniculitis, and perineural inflammation; and direct signs of infection, including the presence of microabscesses and bacterial grains.

### Bacterial grains morphometry

Tissue biopsy sections underwent H&E staining and were subsequently examined using a Motic RED220 digital microscope equipped with a Moticam BTU camera. Imaging was performed at magnification levels of 10x and 40x, employing Motic Images Plus 3.1 software (Motic, China). Bacterial grains were identified according to their distinctive morphological characteristics, with their presence confirmed by a blinded pathologist to mitigate observer bias. For morphometric analysis, all visible grains within the tissue section were manually selected, and their area (μm²), perimeter (μm), and circularity index (calculated as circularity = 4π × area / (perimeter²)) were quantified using the integrated measurement tools of the software, following the provider calibration procedures. Grains that intersected with the image boundaries were excluded to prevent partial measurements. 

### Statistical analysis

Normality was assessed using the Shapiro-Wilk test. Descriptive data are expressed as mean ± standard deviation (SD) or median and interquartile range (IQR), depending on data distribution. Statistical comparisons between groups were performed using one-way ANOVA followed by Tukey’s *post hoc* tests (parametric data) or Kruskal-Wallis with Dunn’s correction (non-parametric data). For histopathological scores, a two-way ANOVA was applied to evaluate the interaction between time points (dpi) and histopathological parameters (e.g., necrosis, microabscess formation), followed by multiple comparisons test. All analyses were conducted in GraphPad Prism 8.0.1 (GraphPad Software Inc., USA), with statistical significance set at *P*<0.05.

## Results

### Clinical evaluation of the chronic mycetoma lesion

**Figure 1:**
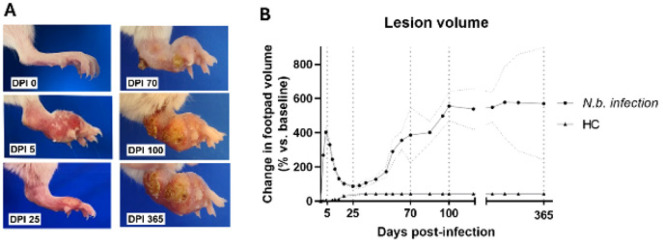
Clinical progression of mycetoma lesion in BALB/c mice infected with *Nocardia brasiliensis*

Progression of the lesion over time was characterized by a trajectory consistent with the natural history of mycetoma lesion patterns (Figure 1a). Clinical progression of mycetoma revealed distinct phases. During the acute phase, localized hyperemia, erythema, swelling, and the formation of a cutaneous nodule in the site of the inoculation, culminated in peak lesion volume by 5 dpi (0.3433 cm^3^ ± 0.0202 cm^3^, 402.80% ± 27.89% of volume change relative to baseline levels, n=18). By 25 dpi, the signs of inflammation subsided (lesion volume 0.1236 cm^3^ ± 0.0057 cm^3^, 73.63% ± 9.36, n=18), though residual induration of the lesion persisted. A resurgence of inflammation began at 30–45 dpi, marked by intermittent edema, serous exudation, and early sinus tract formation.

The chronic phase (70 - 365 dpi) was defined by clinical stability: lesion volume plateaued at 70 dpi (lesion volume 0.3954 cm^3^ ± 0.0241 cm^3^, 349.40% ± 64.55%, n=18) with transient and nonsignificant fluctuations at 100 dpi (lesion volume 0.4253 cm^3^ ± 0.0482 cm^3^, 523.90% ± 74.78%, n=12). Advanced chronicity at 365 dpi (lesion volume 0.3259 cm^3^ ± 0.0246 cm^3^, 508.5% ± 144.4%, n=6) exhibited suppurative abscesses, fistulous tracts, draining sinuses discharging seropurulent material, and macroscopic white grains, and the characteristic deformation of the extremity without signs of pain. A summarizing figure showed these size changes (Figure 1.b). 

It is important to highlight that the analysis conducted at 365 dpi involved only three animals (n=3); three mice were sacrificed due to substantial challenges in mobility and feeding due to the nature of the lesion, like volume increase and deformity of the foot pad, which resulted in a significant loss of body weight (17.23 g ± 0.26 g, percentage of bodyweight loss relative to baseline 25.49% ± 1.94%) compared to the remaining three animals which showed no other signs of distress (22.81 g ± 0.22 g, percentage of bodyweight gain 24.83 % ± 1.11%), approximately at 300 dpi. However, the necropsy data was collected.

**Figure 2:**
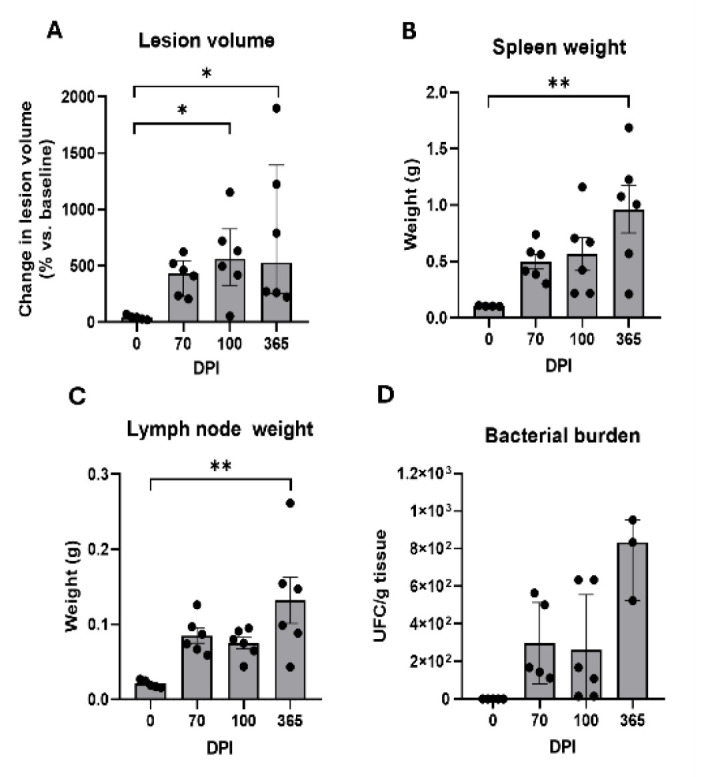
Macroscopic evaluation of local and systemic disease in BALB/c mice infected with *Nocardia brasiliensis*

Detailed findings on mycetoma size, spleen, and lymph node weight, as well as bacterial load of infected tissue at 70 (n=6), 100 (n=6), and 365 dpi (n=6), are presented in Figure 2. The percentage change in the footpad volume exhibited significant variations; the post-hoc analysis indicated a substantial increase in lesion volume at both 100 dpi (*P*=0.0214 vs 0 dpi) and at 365 dpi (*P*=0.0144 vs 0 dpi). In contrast, no progression of the lesion was observed during the chronic phase (Figure 2a). Furthermore, a notable increase in splenic mass was recorded at 365 dpi when compared to uninfected controls (*P*=0.0054) (Figure 2b). Likewise, lymph node weight showed significant differences over time (*P*=0.0032), with the 365 dpi group exhibiting a significant increase in lymph node mass relative to the baseline (*P*=0.0016 compared to 0 dpi), thus indicating persistent antigenic stimuli and host response to this infection (Figure 2c).

*N. brasiliensis *colony forming units are presented in Figure 2d, showing that bacterial burden remained unchanged at 70 (n=6), 100 (n=6), and 365 dpi (n=3), with a slight tendency to increase 365 dpi, which exhibited no statistically significant differences across time points (*P*=0.0995) but a tendency to increase at 365 dpi, indicating that the bacterial burden also reaches a plateau early in chronic infection. Consequently, it was investigated whether the dynamics of the chronic phase are more significantly influenced by adaptive or innate immune cell populations rather than by the pathogen load itself. 

### T cell populations in lymphoid tissues and mycetoma lesions

**Figure 3:**
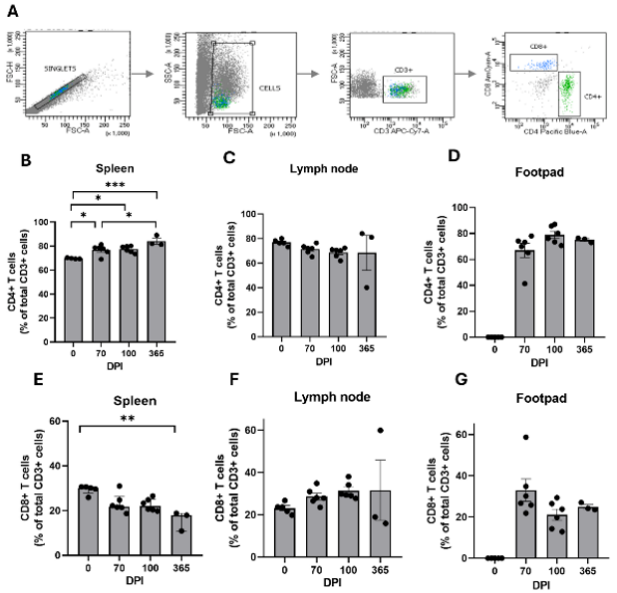
T cell populations in lymphoid tissues and mycetoma in BALB/c mice infected with *Nocardia brasiliensis*

The data was obtained through flow cytometry, where the proportion of CD4+ and CD8+ T cells was evaluated at 70 (n=6), 100 (n=6), and 365 dpi (n=3). Figure 3a demonstrates a statistically significant increase in CD4+ T cells within the spleen across multiple time points (*P*=0.0005), reaching a peak at 365 dpi compared to both 0 dpi (*P*=0.0002) and 70 dpi (*P*=0.0315) (Figure 3b). The values from 70 dpi indicate a notable increase (*P*=0.0315 vs 365 dpi), suggesting a sustained activation of splenic CD4+ T cells over time. In contrast, no significant changes were observed in the population within the popliteal lymph node (*P*=0.8483) (Figure 3c). Although a significant infiltration of CD4+ T cells was present in the mycetoma lesion, no fluctuations were noted across the time points (*P*=0.1307) (Figure 3d). 

Observing CD8+ T cells, a gradual decline was recorded in the spleen by 365 dpi compared to 0 dpi (*P*=0.0049), which was in stark contrast to the increasing trend of CD4+ T cells at the same time point (Fig 3e). Furthermore, CD8+ T cells remained unchanged in both the popliteal lymph node (*P*=0.8486) (Figure 3f) and the mycetoma lesion (*P*=0.1370) throughout the study period, with no CD8+ T cell presence detected at 0 dpi in the mycetoma lesion, despite significant infiltration being observed at chronic time points (Figure 3g). A remarkable finding was that the total T cell CD3+ population in mycetoma represented only a small percentage of the total recorded events (70 dpi, 0.5800%±0.2517%; 100 dpi, 1.179%±0.6728%; 365 dpi, 2.866%±0.7507), and out of this cluster, the CD4+ T cell population was predominant in the mycetoma lesion, comprising 71.59% ± 5.442% at 70 dpi, 78.22% ± 2.707% at 100 dpi, and 75.81% ± 1.062% at 365 dpi of the total T cell population. 

### Myeloid cell populations in lymphoid tissues and mycetoma lesion

**Figure 4:**
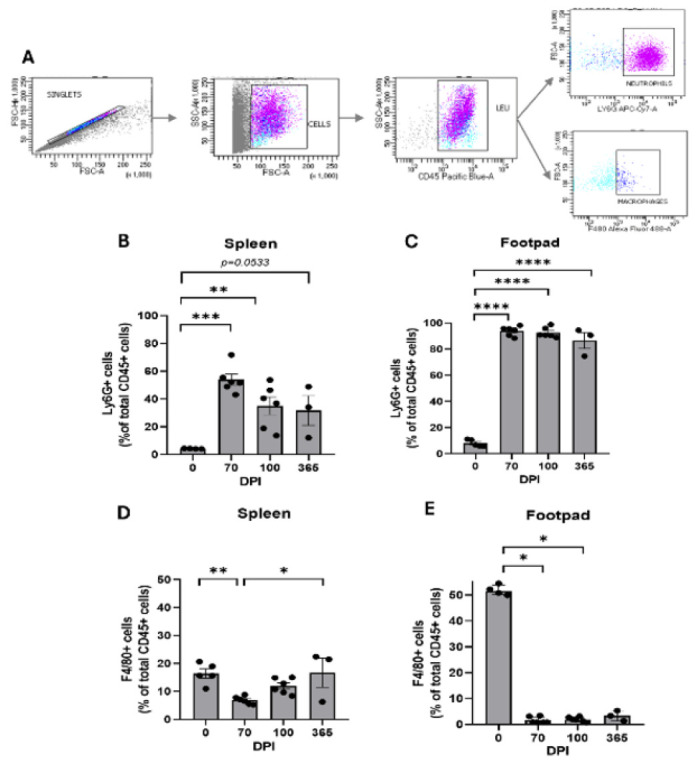
Myeloid populations in lymphoid tissues and mycetoma in BALB/c mice infected with *Nocardia brasiliensis*

Regarding myeloid populations analysis, Ly6G+ cells were classified as neutrophils and F4/80+ cells as macrophages according to the flow cytometry strategy (Figure 4). Focus was placed on the tissues that showed a dynamic response to infection, the spleen, and mycetoma lesions, while the popliteal lymph node was excluded, as it was demonstrated to represent a steady state compartment in this model, analyzed at 70 (n=6), 100 (n=6), and 365 dpi (n=3). In the spleen, a transient surge in neutrophils was observed at 70 dpi (*P*=0.0001 vs 0 dpi) but declined by 365 dpi (*P*=0.0533 vs 0 dpi), with no difference across time points (Figure 4b). In mycetoma, neutrophils dominated the leucocyte infiltrate in all chronic infection time points (0 dpi, 6.667%±1.1705; 70 dpi,95.13%±1.485%; 100 dpi, 90.80%±1.574%, 365 dpi, 90.59±6.050%), compared to no infection (*P*<0.0001) (Figure 4c), with no difference between time points. 

Concerning macrophages, the spleen exhibited an initial reduction at 70 dpi (*P*=0.0069 vs 0 dpi), followed by a resurgence at 365 dpi (*P*=0.0167 vs 70 dpi) (Figure 4d). In the mycetoma lesion, macrophages precipitously declined during chronic infection compared to no infection (*P*=0.0148 vs 70 dpi, *P*=0.0407 vs 100 dpi) (Figure 4e). These findings indicate compartmentalized immune coordination at the infection site, characterized by a predominance of CD4+ T cells and neutrophils. 

This cellular arrangement facilitates the local containment of the pathogen, resembling both acute and chronic infections. Thus, it is essential to delineate the cytokine networks that facilitate the cellular migratory response. 

### Serum cytokines

**Figure 5:**
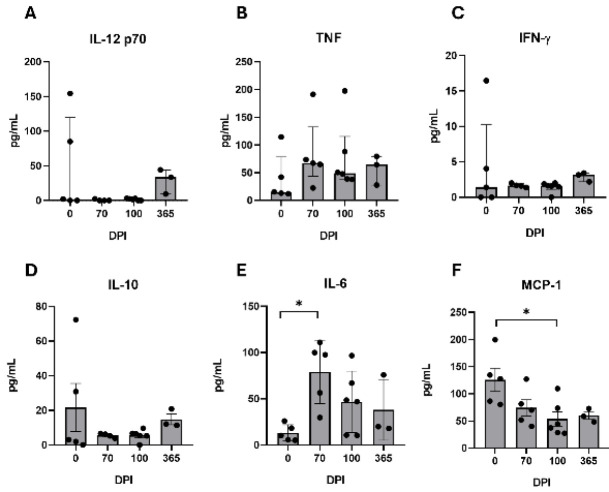
Serum cytokines profile from BALB/c mice infected with *Nocardia brasiliensis*

Analysis of serum cytokine dynamics during chronic infection revealed no significant variations in the concentrations of the bioactive form of IL-12 (IL-12p70), TNF, IFN-γ, or IL-10 at 70 (n=6), 100 (n=6), and 365 dpi (n=3). This suggests minimal engagement of Th1 polarization or regulatory cytokines during the chronic phases of the infection (*P*=0.1053, *P*=0.3670, *P*=0.2835, *P*=0.3970) (Figure 5a-d), indicating an atypical chronic immunopathology, that likely allows bacterial persistence. Serum levels of IL-6 were elevated at 70 dpi (*P*=0.0132 vs 0 dpi), following a gradual decline towards 365 dpi (Figure 5e), consistent with the recruitment of neutrophils to the site of infection, reminiscent of an acute inflammatory pattern. MCP-1 levels exhibited a progressive downward trend over time, decreasing significantly by 100 dpi (*P*=0.0208 vs 0 dpi) (Figure 5f), indicating a possible decline in monocyte recruitment over time, as evidenced by the local and systemic decrease in macrophages. These findings prompted us to search for histological evidence that indicates acute tissue inflammation and bacterial persistence.

### Histopathology of the mycetoma lesion

**Figure 6:**
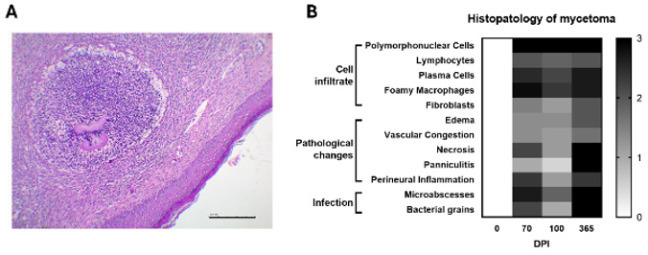
Histopathology of mycetoma lesion in BALB/c mice infected with *Nocardia brasiliensis*

Tissue biopsies were analyzed and exhibited the characteristic bacterial grain morphology. These formations present a distinctive shape with a spiculated periphery. They are situated at the center of a microabscess, which is characterized by a significant infiltration of PMN cells. The outermost layer of the abscess is encircled by a ring of mononuclear cells, primarily consisting of giant cells and foamy macrophages. This structural arrangement is localized within the dermis (Figure 6a). Pathological findings in the tissue biopsies were reported with a histological score for analysis at 70 (n=6), 100 (n=6), and 365 dpi (n=3). These results were aligned with those shown by flow cytometry, which revealed a high number of PMN cells infiltrating the lesion and a small infiltrate of mononuclear cells at all time points. Also, greater tissue damage was found a year after the start of infection, like necrosis (*P*=0.0302 vs 100 dpi), panniculitis (*P*<0.0001 vs 70 dpi and 100 dpi), perineural inflammation (*P*=0.0302 vs 100 dpi), the formation of microabscess (*P*=0.0302 vs 100 dpi), and bacterial grains (*P*<0.0001 vs 100 dpi), reflecting a mixed acute-chronic inflammatory response that progressed significantly over time (Figure 6b). Importantly, these pathological changes—critical drivers of tissue destruction—were undetectable by external evaluation or lesion volume measurements, underscoring the necessity of histological assessment to capture the underlying complexity of chronic *N. brasiliensis *infection fully. 

### Bacterial grain morphology

**Figure 7:**
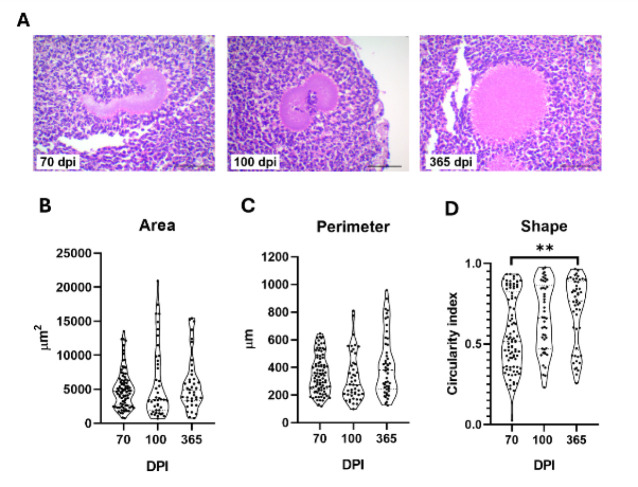
Microcolony shape shifts in chronic infection in BALB/c mice infected with* Nocardia brasiliensis*

Through histological analysis, a notable difference in bacterial grain morphology over time was noted, shifting from a “club” shape at 70 dpi to a more circular form by 365 dpi, prompting us to conduct a morphometric analysis (Figure 7a) at 70 (n=6), 100 (n=6), and 365 dpi (n=3). This analysis aimed to evaluate the area, perimeter, and shape of bacterial grains to investigate their structural adaptations during chronic infection from a single tissue section sample of the mycetoma at 70 dpi (n=6, with a total of 90 individual bacterial grains), 100 dpi (n=6, 46 bacterial grains), and 365 dpi (n=3, 50 bacterial grains) (Figure 7b). Although no statistically significant differences were detected in perimeter (*P*=0.0838) or area (*P*=0.5420) across the time points, the shape displayed a notable evolution towards circularity during the course of chronic infection. The extent to which the shapes resemble a perfect circle was quantified using the circularity index. (1.0 signifies a perfect circle); the circularity index was 0.5173 (0.3584-0.8234) at 70 dpi, increased to 0.6622 (0.4698-0.8633) at 100 dpi, and reached 0.7676 (0.4619-0.8897) at 365 dpi. This indicates a significant increase in circularity over time (70 vs 100 dpi, *P*=0.0504; 70 vs 365 dpi, *P*=0.0044). This transition to a more circular grain morphology in chronic mycetoma may indicate a stress-adapted bacterial phenotype with implications for disease progression and immune evasion.

### Key findings

Chronic mycetoma induced by *N. brasiliensis* in mice revealed stable bacterial burden and lesion volume after 70 dpi, despite progressive tissue necrosis and panniculitis undetectable by external examination. Chronic lesions were marked by sustained neutrophil dominance (>90% infiltration), systemic CD4⁺ T cell expansion in the spleen, and a cytokine shift toward IL-6 elevation, suggesting Th17 polarization alongside declining MCP-1. Bacterial grains underwent structural remodeling, transitioning from club-shaped to circular morphologies, while granuloma-like fibrosis and persistent neutrophilic activity drove tissue destruction. Crucially, disease severity uncoupled from microbial load in late stages, with IL-6-driven inflammation and Th-CD4⁺ responses emerging as central pathological drivers.

## Discussion

The progression of mycetoma caused by *N. brasiliensis* in our murine model exemplifies a complex interplay between pathogen persistence and host immune dynamics, marked by unresolved inflammation that contributes to chronicity. This highlights the utility of this model in investigating chronic bacterial infections that result in granulomatous lesions comparable to those seen in tuberculosis and leprosy and involving less pathogenic bacteria in non-immunocompromised humans (7, 25). The triphasic trajectory of lesion development—marked by an acute inflammatory peak, transient remission, and late resurgence—suggests an immune-driven pathology that evolves independently of bacterial burden, which plateaus early in chronicity. Additionally, the presence of lesions, accompanied by notable lymphoid hyperplasia (e.g., splenomegaly), suggests a persistent state of systemic immune activation. However, the local immune environment at the site of infection exhibited a marked contrast: neutrophils predominated in the leukocyte infiltrate, while macrophages experienced a significant decline. T cells, primarily of the helper subset, constituted a minor yet stable component. This compartmentalization suggests a failed resolution mechanism without transitioning from acute neutrophilic inflammation to macrophage-mediated resolution, favoring chronicity over clearance. This dissociation between clinical inflammation and pathogen load parallels observations in other chronic infections caused by acid-fast bacteria, like *Mycobacterium tuberculosis *and* Mycobacterium leprae*, where dysregulated immunity outweighs direct microbial destruction (26, 27). 

The absence of substantial Th1-polarizing cytokines (IL-12p70, IFN-γ) in serum is particularly noteworthy, contrasting with established chronic bacterial infections where these mediators are essential for local containment. Previous investigations have also documented that IFN-γ was detectable in only 30% of the cells within the *N. brasiliensis *mycetoma microabscess by 90 dpi, while TNF-α was undetected throughout the infection period (28). Furthermore, the noted increase in IL-6, previously recorded in all macrophages and lymphocytes adjacent to the microabscess, as well as in skeletal muscle (28), along with the progressive decrease in MCP-1, suggests a neutrophil-driven inflammatory response. A key feature of the transition from acute to chronic inflammation is the recruitment of monocytes to the inflamed area. The IL-6/IL-6Rα complex promoted due to substantial stromal cell damage, can activate endothelial cells to secrete IL-8 and MCP-1 while also inducing adhesion molecules (29). In our model, it was observed that IL-6 secretion is evident in serum and is sustained throughout the chronic phase; however, it is interesting to note that MCP-1 levels do not exhibit an increase, thus indicating a halt in the acute phase that does not confer antimicrobial advantages, paralleling sterile chronic inflammatory conditions such as rheumatoid arthritis (30).

Histopathological findings highlight the silent progression of tissue destruction, allowing the slow spread of infection to local tissues, including the subcutaneous tissue. The host immune response plays a crucial role in ongoing inflammation and in keeping bacteria at bay to avert systemic infection, although it simultaneously hinders resolution. Despite stable lesion volumes evaluated externally in the chronic stage, the presence of advanced necrosis and panniculitis formation at 365 dpi reveals a disconnect between external clinical metrics and the underlying pathology, supporting our notion that acute and chronic inflammation coexists in the microenvironment. 

The classical actinomycetoma microabscess architecture— packed microcolonies, surrounded by neutrophil-rich cores further encircled by mononuclear cells, mainly foamy cells and giant multinucleated cells with fibroblast proliferation with production of collagen to locally prevent bacterial systemic dissemination—mirrors granulomatous structures seen in human actinomycetoma (28, 31–33). It is suggested that the arrangement of microcolonies enables bacteria to avoid immune system elimination, including resistance to phagocytosis (34). The morphological evolution of *N. brasiliensis* microcolonies towards a circular form during chronicity, reflecting a stress-adapted phenotype, may enhance this evasion, potentially through structural adaptations similar to biofilm formation, of which this bacterium has already proven to be a stronger producer (35). Biofilm formation in other infections produced by *Nocardia spp.* is related to central venous catheter-associated bacteriemia (36). 

It has been proposed recently that the computational analysis of histological images from mycetoma lesions could enhance diagnosis and thereby facilitate appropriate treatment based on trained algorithms that can discriminate the etiological pathogen through grain segmentation analysis (37). However, it was demonstrated that the microcolony tends to change its shape over time, indicating that morphology may reflect the age of the colony itself rather than the pathogen identity. Thus, it is emphasized that, in addition to the recognized gold standard of grain culture for pathogen identification (31), histopathological analysis of the lesion and serological tests (16, 38) must be performed to evaluate the local extent of the infection, tissue damage, and host immune response. 

In line with this model, prior investigations into regulatory T cells (CD4+ FOXP3+) revealed their transient elevation exclusively during the acute phase (7 dpi) in mycetoma tissue and spleen, coinciding with reduced IFN-γ, and elevated TGF-β, IL-10, and bacterial load. However, in the chronic phase (60 dpi), Treg populations and IL-10 production declined to negligible levels (23, 28). These dynamics suggest that regulatory T cell-mediated immunosuppression or IL-10 overproduction cannot fully account for chronic mycetoma progression. Instead, the sustained neutrophilic inflammation and unsuccessful adaptive immunity observed here dominate the chronic phase.

Additionally, previous findings have shown that *N. brasiliensis* orchestrates a temporal immune shift: an early Th17 surge (3 dpi) primes neutrophil recruitment, followed by Treg expansion (7–15 dpi) that suppresses IFN-γ, thereby enabling transient bacterial control. However, by chronic stages (≥60 dpi), Tregs and IL-10 wane, while IL-6 persists, creating a void in regulatory restraint and propelling a Th17-skewed milieu (23). This IL-6-dominated environment favors neutrophil survival and disrupts macrophage-mediated resolution, as evidenced by the decline in MCP-1. Concurrently, the absence of IFN-γ and IL-12p70 cripples Th1 responses, leaving the host reliant on neutrophilic inflammation—a response insufficient to clear N. brasiliensis grains but sufficient to perpetuate tissue damage. Thus, the transition from acute immunosuppression to chronic neutrophil dominance reflects a pathogen-driven strategy to exploit immune dysregulation, where waning Treg activity unmasks unresolved Th17 inflammation, and the lack of Th1/Th17 balance locks the host into a futile cycle of granulomatous containment and necrosis.

Chronic mycetoma, characterized by neutrophilic infiltration and reduced mononuclear cell engagement, aligns with patterns seen in other chronic infections and inflammatory disorders where acute-phase responses fail to resolve. For instance, in patients with cystic fibrosis who have lung infections due to *Pseudomonas aeruginosa, *bacterial biofilms are responsible for persistent neutrophil recruitment through IL-8 signaling. In this cited study, neutrophils fail to clear the pathogen due to oxidative burst dysfunction and biofilm resistance (39). In agreement, the *N. brasiliensis* grain structure in mycetoma may contribute to evading phagocytosis and perpetuating neutrophil influx despite chronicity. This shared mechanism highlights how pathogen adaptations subvert neutrophil-mediated bacterial clearance, creating a deleterious cycle of tissue damage and inflammation. 

In other chronic intracellular bacterial infections, such as lepromatous leprosy and type 2 reactions (erythema nodosum leprosum), it has been shown that neutrophilic microabscesses occur in the chronic stages, driven by *M. leprae*-induced immune evasion (40, 41). The absence of Th1-polarizing cytokines and macrophage dysfunction are similar to our findings in mycetoma, where IL-6 dominates over IL-12p70 and IFN-γ, favoring neutrophil chemotaxis and polarization to a Th17 phenotype while impairing macrophage and Th1-cell responses. Likewise, chronic osteomyelitis caused by *Staphylococcus aureus* biofilms exhibits neutrophil-dominated lesions with sparse lymphocytes (42), akin to mycetoma’s lymphoid hyporesponsiveness. These parallels underscore a broader theme: chronic infections often evolve into a pathological stalemate, where neutrophils amplify inflammation without resolving the infection.

Tuberculosis in immunocompromised also exemplifies this phenomenon. In cases of HIV-associated tuberculosis, necrotic granulomas form neutrophil-rich caseous foci due to impaired macrophage activation and T-cell exhaustion, ultimately resulting in liquefactive necrosis (43). Similarly, in the present work, mycetoma’s necrotic progression and perineural inflammation suggest that neutrophil-derived proteases and reactive oxygen species exacerbate tissue damage, overwhelming mononuclear cell repair mechanisms. Dysregulated cytokines promote this mechanism: elevated IL-6 promotes neutrophil survival, while declines in MCP-1 reduce monocyte recruitment, creating an imbalance that sustains neutrophilic influx.

In human patients with hidradenitis suppurativa, a predominant neutrophilic infiltration is observed, often accompanied by recurrent abscesses (44). Like mycetoma, hidradenitis suppurativa lesions show minimal lymphocyte infiltration and fail to transition to macrophage-mediated resolution, suggesting shared pathways of immune dysregulation.

The persistence of neutrophil-dominated inflammation, rather than a transition to macrophage-mediated resolution, in our murine mycetoma model, may reflect the involvement of multifactorial mechanisms. First, the formation of microbial granules likely acts as a physical barrier, impairing macrophage phagocytosis while sustaining neutrophil recruitment through continuous chemokine release (e.g., IL-8/CXCL1) (29). Pathogen-specific virulence factors, such as catalase or anti-oxidant enzymes produced by *N. brasiliensis*, could further neutralize neutrophil-derived reactive oxygen species, prolonging microbial survival and perpetuating neutrophilic inflammation (13). Second, dysregulation of pro-resolving lipid mediators (e.g., resolvins) or an imbalance in cytokine profiles (e.g., elevated IL-1β/TNF-α vs deficient IL-10) may disrupt macrophage polarization toward anti-inflammatory or reparative phenotypes (24, 29). Third, chronic antigen exposure could induce CD4+ T cell and macrophage exhaustion, as observed in other persistent infections, reducing their capacity to clear granules or modulate inflammation (45, 46). Additionally, perilesional fibrosis, a hallmark of advanced mycetoma, may physically restrict macrophage infiltration into the infection site (28). These mechanisms, consistent with human mycetoma pathophysiology, highlight the challenges in achieving immune resolution and underscore the need for therapies targeting both pathogen clearance and host immunomodulation. Future studies profiling temporal cytokine dynamics or macrophage functional states in this model could further elucidate these transitions.

The findings collectively point out that chronic mycetoma is maintained through a maladaptive immune equilibrium. Within the microabscesses that characterize mycetoma, where neutrophils predominate in the inflammation site, coinciding with diminished macrophage and T cell numbers, facilitate bacterial persistence. These observations challenge traditional models of chronic bacterial infections and underscore the necessity for dual-target therapies that simultaneously address both pathogen persistence and host immunopathology. To disrupt this maladaptive immune equilibrium, new therapeutic strategies must include biofilm-targeted agents, such as matrix-degrading enzymes (e.g., dispersin B or DNase I), to dismantle bacterial aggregates (47, 48). Additionally, immunomodulators such as IL-6/JAK-STAT inhibitors (tocilizumab) can be employed to attenuate neutrophil-driven inflammation (49, 50). Simultaneously, checkpoint inhibitors, like anti-PD-1 monoclonal antibodies, may reinvigorate exhausted T cells (51). In addition, therapies targeting macrophages (GM-CSF or PPAR-γ agonists) can potentially restore phagocytic clearance (52, 53). Additionally, it is essential to emphasize the importance of therapeutic vaccines that can complement the proposed therapeutic approaches. Vaccine development should contain antigens present on bacterial aggregates, paired with Th1-polarizing adjuvants, like CpG oligonucleotides, to redress the identified cytokine imbalance and enhance adaptive immunity (54). These strategies provide a translational framework to dismantle mycetoma’s self-perpetuating cycle of inflammation and bacterial persistence.

(A) Representative images of mycetoma in the BALB/c mice footpad. (B) The lesion progression is represented as a percentage of change in the volume of the footpad. HC= Healthy controls. The data represent the mean of six mice per group ± the SEM.

(A) The mycetoma percentage of volume change was evaluated at defined time points. (B) Spleen and (C) lymph node weight was evaluated, as well as (D) bacterial burden in infected tissue. The data represents the median ± IQR for A and D, and the mean ± SEM for B and C. DPI= Days post infection; UFC= Colony forming units. *P< 0.05, **P< 0.01.

(A) The representative flow cytometry gating strategy for Th CD4+ and CD8+ Tc T cells. (B) Spleen, (C) lymph node, and (D) infected footpad-mycetoma CD4+ Th percentages were calculated relative to the total lymphocytes evaluated, as well as (E) spleen, (F) lymph node, and (G) mycetoma CD8+ Tc percentages using the mentioned strategy. The data represent the mean ± SEM for B-D and F-G, and median ± IQR for E. DPI= Days post infection. *P <0.05, **P<0.01, ***P<0.001, ****P<0.0001.

(A) The representative flow cytometry gating strategy for Neutrophils and macrophages (B) Spleen, and (C) infected footpad-mycetoma neutrophil percentages were calculated relative to the total leucocytes, as well as (D) spleen and (e) mycetoma macrophages percentages using the mentioned strategy. DPI= Days post infection. The data represents the mean ± SEM for B-D, and median ± IQR for E. *P<0.05, **P < 0.01, ***P<0.001, ****P<0.0001.

(A-F) Concentration of serum cytokines analyzed by CBA. DPI= Days post infection.The data represent median ± IQR for A-C, and the mean ± SEM for D-F. *P<0.05.

(A) Representative images of microabscess from the mycetoma in the BALB/c mice footpad. Bar=200 µm. (B) The heathmap of representative pathological features in infected tissue. DPI= Days post infection. The data represent the mean histological score of six mice per group.

(A) Representative images of microcolonies from the mycetoma in the BALB/c mice footpad. Bar=50 µm. (B) The area, perimeter, and circularity (4π × area / (perimeter²) of each microcolony found in the complete section were measured. DPI=Days post infection. The data represent median ± IQR for B-D. **P<0.01.

## Conclusion

This study demonstrates that chronic *N. brasiliensis*-induced mycetoma in the murine model described here is driven by a maladaptive immune equilibrium, where persistent neutrophil infiltration is probably related to excessive secretion of IL-6 and CD4+ T cell responses that sustain inflammation independently of bacterial burden, leading to progressive tissue damage despite clinical stability. The uncoupling of pathogen load from disease severity, alongside the morphological evolution of bacterial grains, suggests that immune dysregulation—not microbial proliferation—becomes the primary driver of pathology in late-stage infection.

## Declaration

During the preparation of this work, the authors utilized Grammarly to enhance readability and language. After using this tool, the authors reviewed and edited the content as needed and take full responsibility for the content of the publication.

## Limitations of the Study

The limitations of this study, including the reduced cohort size at 365 dpi and the inherent constraints of the murine model, caution against direct extrapolation to human mycetoma. Further investigation is suggested.
